# Overlapping yet dissociable contributions of superiority illusion features to Ponzo illusion strength and metacognitive performance

**DOI:** 10.1186/s40359-024-01625-9

**Published:** 2024-03-01

**Authors:** Daisuke Matsuyoshi, Ayako Isato, Makiko Yamada

**Affiliations:** 1 Institute of Quantum Life Science, National Institutes for Quantum Science and Technology, 4-9-1 Anagawa, Inage, Chiba, 263-8555 Japan; 2 Department of Functional Brain Imaging Research, Institute of Quantum Medical Science, National Institutes for Quantum Science and Technology, 4-9-1 Anagawa, Inage, Chiba, 263-8555 Japan; 3 Araya Inc., 1-11 Kanda-sakumacho, Chiyoda, Tokyo, 101-0025 Japan; 4 Faculty of Humanities, Saitama Gakuen University, Saitama, 333-0831 Japan

**Keywords:** Machine learning, Superiority illusion, Visual illusion, Metacognition, Bayesian estimation, Ponzo illusion

## Abstract

Humans are typically inept at evaluating their abilities and predispositions. People dismiss such a lack of metacognitive insight into their capacities while even enhancing (albeit illusorily) self-evaluation such that they should have more desirable traits than an average peer. This superiority illusion helps maintain a healthy mental state. However, the scope and range of its influence on broader human behavior, especially perceptual tasks, remain elusive. As belief shapes the way people perceive and recognize, the illusory self-superiority belief potentially regulates our perceptual and metacognitive performance. In this study, we used hierarchical Bayesian estimation and machine learning of signal detection theoretic measures to understand how the superiority illusion influences visual perception and metacognition for the Ponzo illusion. Our results demonstrated that the superiority illusion correlated with the Ponzo illusion magnitude and metacognitive performance. Next, we combined principal component analysis and cross-validated regularized regression (relaxed elastic net) to identify which superiority components contributed to the correlations. We revealed that the “extraversion” superiority dimension tapped into the Ponzo illusion magnitude and metacognitive ability. In contrast, the “honesty-humility” and “neuroticism” dimensions only predicted Ponzo illusion magnitude and metacognitive ability, respectively. These results suggest common and distinct influences of superiority features on perceptual sensitivity and metacognition. Our findings contribute to the accumulating body of evidence indicating that the leverage of superiority illusion is far-reaching, even to visual perception.

## Introduction

Contrary to our naïve belief, humans often do not have accurate insight into themselves. The metacognitive capacity to assess self-made decisions or personal abilities varies substantially across individuals, typically not reaching the full information theoretically available to an individual [1, 2]. Despite the predominant lack of metacognitive insight, people often regard themselves as competent and having more desirable traits than an average peer [3–5]. At first glance, this superiority illusion (SI) appears as a metacognitive ability defect. However, evidence suggests that SI helps maintain a healthy mental state [3, 4, 6, 7], self-esteem [8, 9], and life satisfaction [9, 10], except for overly optimistic self-evaluations [11–14]. Therefore, rather than a defect, SI is likely to be a self-serving cognitive bias with a myriad of psychological benefits.

Numerous studies have shown that SI occurs in various domains [15–17] with cross-cultural robustness [18–21], indicating its universal and fundamental contributions to human behavior. However, the scope and range of how SI influences perception remain unclear. As cognitive style underlies how people perceive, think, solve problems, learn, and relate to others [22], SI also likely exerts its heuristics, even over perception, by biasing cognition and decision-making toward illusory ones. For example, field-dependence/independence is among the best-known cognitive styles, where people who exhibit field-dependence tend to use a holistic or contextual approach to perceive the world [23]. Field-dependent people are known to be inept at absolute size estimation [24] and various visuospatial tasks [25] and are susceptible to the Ponzo illusion [26], perhaps because of their greater reliance on visuospatial contexts, such as integrating an object within its surroundings. Although Zhang [27] claimed that the field-dependence/independence construct represents a perceptual ability rather than a cognitive style, later studies have demonstrated that cognitive styles represent behavioral heuristics that govern across multiple levels of information processing, from perceptual ability and metacognition to personality traits and social skills [28–31].

**Fig. 1:**
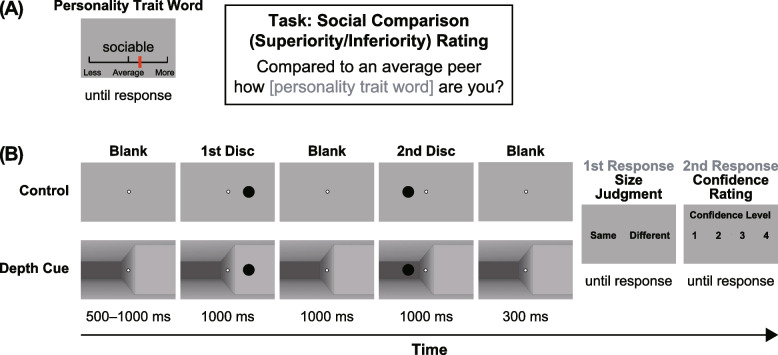
Experimental paradigm. **A** Schematic presentation of the superiority rating task. Participants indicated how personality trait words described them compared to an average peer using a sliding scale. **B** Schematic presentation of the Ponzo illusion task. Participants were required to indicate whether the two discs were the same size (1st response) and then rate their confidence (2nd response). The size of the fixation point is exaggerated for illustration purposes

In this study, we used hierarchical Bayesian estimation and machine learning of signal detection theoretic (SDT) measures to understand how SI influences the Ponzo illusion. As retinal images are inherently ambiguous (e.g., a distant large or a closer small object could invoke the same retinal projection), human vision resolves ambiguities by biasing neural activities based not only on visual contexts but also on knowledge or beliefs [32, 33]. We hypothesized that visual illusions are a powerful window into how we incorporate various sources and create best-bet predictive hypotheses of objects and situations for optimal, adaptive behavior while handling uncertainties. We chose the Ponzo illusion as a visual stimulus [34] since it must be mediated by feedback projections from higher areas and is prone to the top–down control [35, 36]. Compared to other visual illusions established only by lateral connections in the primary visual cortex [37–39], these characteristics of the Ponzo illusion are desirable for our study investigating the effects of top–down, illusory cognitive bias. To examine Ponzo illusion magnitude perception and its metacognition in the SDT framework, unlike a typical experiment using a method of adjustment or constant stimuli, we asked participants whether the two stimuli were the same or different (same/different task) and to rate their metacognitive confidence about the perceptual decision (confidence rating task) (Fig. 1). Although a demanding task that leads to inefficient behavioral performance (e.g., visual illusion) often prevents us from estimating reliable metacognitive ability [1, 40], hierarchical Bayesian estimation allows for accurately estimating metacognitive measures even when low sensitivity is expected because of illusory percepts [41].

Moreover, we combined principal component analysis (PCA) and cross-validated regularized regression (relaxed elastic net) to create prediction models for the Ponzo illusion magnitude and metacognitive performance from SI rating data. This combined machine learning approach allowed us to uncover the models’ latent architecture by examining the weighted total feature importance (the product of SI PCA loadings and prediction model feature importance). Our approach focuses on effectively extracting latent information in the data rather than simply creating prediction models, thereby enabling us to gain an in-depth understanding of behavioral correlations by unveiling differential influences of SI features on Ponzo illusion perception and metacognition.

## Materials and methods

### Participants

All participants were recruited from a volunteer recruitment website managed by the National Institutes for Quantum Science and Technology. Exclusion criteria included the participant’s unwillingness to participate, history of neurological or psychiatric conditions, and inability to communicate in Japanese. Thirty-seven males participated in this study (mean age: 23.3 ± 3.1 years [1 SD]; range: 20–32 years). All had normal or corrected-to-normal vision and reported no known neurological or psychiatric conditions. We did not perform a power analysis to determine the sample size. We heuristically stopped data collection as we reached a sample size of approximately double the typical, old-fashioned number of 20 participants.

### Stimuli and procedure

We presented stimuli using E-Prime 2.0 (Psychology Software Tools, PA, USA). Participants viewed stimuli on a 24-inch LCD monitor at a distance of 60 cm. We presented all stimuli on a gray background.

#### Superiority rating task

We successively presented personality trait words on the center of the screen with a visual analog scale (VAS) on the bottom (Fig. 1A). We asked participants to rate the extent to which each personality trait word would describe them by comparing themselves with an (imaginary) average peer using a VAS with a step of 0.05 (score ranges from −1 [much less than the average] through 0 [approximately the same as the average] to 1 [much more than the average]). We used 26 desirable, 26 undesirable, and eight filler words from previous studies [5, 42] in randomized order across the participants. Undesirable word scores were reverse-coded. Scores above zero indicate the subjective superiority of the participants compared to an average person (and vice versa). There were no exclusion criteria based on participant’s ratings.

#### Ponzo illusion task

We used a black disc (4.6 to 6.7° diameter, randomized across trials) presented at 8.8° to the left and right of the fixation point centered on the screen as a stimulus to measure the Ponzo illusion (Fig. 1B). The experiment displayed two background image conditions: discs presented on a uniform gray background or a 3D-textured image containing linear-perspective, pictorial depth cues (control and depth cue conditions, respectively).

Each trial comprised the following steps: presentation of a fixation point (500–1000 ms, randomized across trials) followed by a black disc on one side (1000 ms), blank screen (1000 ms), a black disc on the other side (1000 ms), blank screen (300 ms), and two response displays. First, we asked the participants to judge whether the two discs were the same size by pressing a corresponding response pad. Second, the participants had to rate their confidence for the first decision by pressing a corresponding key on a scale of 1 (very unconfident) to 4 (very confident). It is worth mentioning that discs were sequentially, but not simultaneously, presented to produce the Ponzo illusion in our task. Thus, mnemonic components were involved in our Ponzo illusion task; however, Shen et al. [43] found a comparable magnitude of illusion between sequentially and simultaneously presented versions with significant correlation, indicating similar (or identical) mechanisms governing both presentation conditions.

The participants carried out 320 trials, where the “distant” disc was equal to (128 trials), 20% smaller (128 trials), 5% smaller (32 trials), and 5% larger (32 trials) in diameter than the other disc. The 5% larger/smaller sets (32 + 32 trials) represented filler trials and were not analyzed further. Thus, further analyses included the remaining 256 trials (128 + 128 trials). Half of the 320 trials were performed under depth cues, and the other half under control conditions. In the case of the depth cue conditions, the left wall was apparently “close” on half of the trials, and the right wall was apparently “close” on the other half. We always presented the first disc on the “close” side of the wall. Due to the uniform background, no markedly “distant” or “close” disc could be distinguished under the control (but not the depth cue) conditions. The trial order was pseudo-randomized across the trials with the constraint that all conditions appeared in every 40 trials. The participants took a few minutes break after performing 160 trials. There were no exclusion criteria based on participant’s behavioral performance.

### Estimation of SDT measures

To estimate metacognitive efficiency, we computed log(meta-*d’*/*d’*), where *d’* is an SDT measure of type 1 first-order sensitivity (i.e., *perceptual* sensitivity) and meta-*d’* is a measure of type 2 *metacognitive* sensitivity [1], representing a measure of the ability to distinguish between correct and incorrect judgments. Meta-*d’*/*d’*, also called the M-ratio, is a measure of metacognitive *efficiency*, compensating for the intrinsic correlation between meta-*d’* and *d’*. Meta-*d’* equal to *d’* (i.e., M-ratio = 1 and log M-ratio = 0) represents that the observer is metacognitively “optimal”, using all the available information for the type 1 task to the type 2 task. However, people are typically not fully aware of the accuracy of a decision; observers often display metacognitive *inefficiency* (i.e., M-ratio < 1 and log M-ratio < 0) [44]. In contrast, observers occasionally exhibit *superefficiency* (i.e., M-ratio > 1 and log M-ratio > 0) in that they seemingly use more information than the theoretical maximum [45, 46]. Although superefficiency is not well understood, the nonoptimal metacognition (i.e., either inefficiency or superefficiency) implies (at least partially) distinct mechanisms for first-order decisions and confidence ratings.

We performed hierarchical Bayesian estimation of log M-ratio using Markov chain Monte Carlo sampling (3 chains of 10,000 samples and 1,000 burn-in samples) to incorporate within- and between-subject uncertainty [41]. The hierarchical Bayesian approach allows for recovering accurate metacognitive efficiency estimates from confidence ratings even at low *d’* values, where commonly used alternatives fail. This benefits our Ponzo illusion task with an inherently low perceptual sensitivity (i.e., illusion leads to poor discrimination performance). We performed statistical analyses on the log M-ratio (instead of the M-ratio) to ensure that a unit of distance along an axis represents an equal weight relative to the optimal value of meta-*d’*/*d’* = 1 [41, 47].

Type 1 SDT parameters (*d’* and criterion *C*) were also estimated along with this hierarchical Bayesian framework, but the estimated values are exactly identical to conventional, non-Bayesian methods. We estimated meta-*C*, a criterion measure for type 2 decision, using maximum-likelihood estimation [1]. *C* represents a measure of response bias in first-order decisions, and meta-*C* represents a measure of response bias in metacognitive judgments.

### Machine learning model using relaxed elastic net

We created a prediction model using a machine learning technique to examine which superiority rating items best explain each SDT parameter estimate of the Ponzo illusion. We performed a relaxed elastic net, a two-step elastic net regression similar to a relaxed Lasso [48]. Relaxed elastic net regression creates a regularized regression model by performing variable (superiority rating item) selection using the standard elastic net [49] and then determines weight coefficients for the selected variables using ridge regression. This procedure attenuates overfitting and multicollinearity by shrinking variance and results in more reliable estimates than conventional linear regression using ordinary least squares. We created two models: one to predict *d’* and another to predict log M-ratio from 52-item superiority ratings. All variables included in the models were standardized to have zero mean and one variance. We performed a relaxed elastic net regression with leave-one-sample-out cross-validation (LOOCV) that uses grid search to find the optimal hyperparameters. We used $\mathrm{\alpha }\in [0.1, 1.0]$ (a hyperparameter controlling the trade-off between the L1 and L2 penalties) with a step of 0.1 and $\uplambda \in {10}^{[-3, 3]}$ (a regularization hyperparameter) with a step of 2/33 in the initial elastic net, then zero α and the best-tuned λ (from the initial elastic net) to optimize the weight vector of the selected items in the following ridge regression. This two-step procedure effectively reduces the dimensionality of the superiority rating items related to the Ponzo illusion SDT parameter estimates through variable selection while providing more optimal weight estimates than standard elastic net regression [50].

### PCA

We performed a PCA with singular value decomposition on 52-item superiority ratings to estimate latent SI dimensions. We performed a parallel analysis using unweighted least squares to find an optimal number of PCs [51]. Next, to examine the relationship between model-selected superiority rating items and SDT parameter estimates, we calculated an index called weighted total feature importance, representing the relative contribution of each PC to each model by taking the matrix product of feature importance and PCA loadings. Higher (absolute) values indicate a higher contribution of that particular PC to the prediction model. Moreover, we examined correlations between PC scores and SDT parameter estimates to confirm the generic relationship between superiority rating PCs and SDT parameter estimates.

### Statistical inference

We set the statistical thresholds at α = 0.05 for superiority ratings, type 1 SDT measures (*d’* and *C*), and meta-*C* and at the 95% highest density interval (HDI) of posterior distributions for group-level hierarchical Bayesian type 2 SDT parameter estimates (M-ratio and log M-ratio). To accurately capture the effects of the Ponzo illusion, we calculated between-condition differences for the SDT parameter estimates (depth condition − control condition). A negative difference value indicated a higher Ponzo illusion magnitude (*d’*), a more liberal criterion under the illusion (*C*), a more liberal metacognitive criterion under the illusion (meta-*C*), or lower illusion-induced metacognitive performance (M-ratio and log M-ratio). We used parameter estimates from single-subject Bayesian model fits for correlation and individual difference analyses. We assessed correlations using Spearman’s rho and set the significance threshold at α = 0.05.

## Results

### Superiority rating

**Fig. 2:**
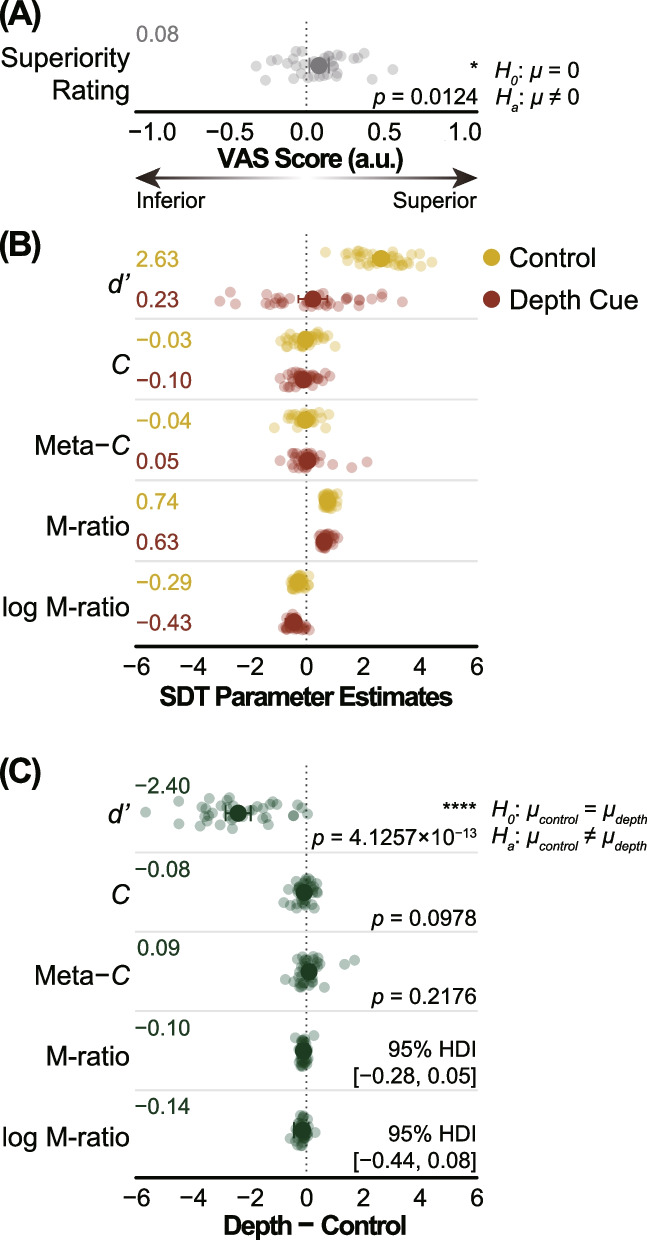
Behavioral results. **A** Superiority rating score. **B** Signal detection theoretic (SDT) parameter estimates for the Ponzo illusion task. **C** SDT parameter estimates differences (between depth cue and control conditions) for the Ponzo illusion task. In **A**-**C**, transparent dots represent individual data points (superiority rating, *d’*, *C*, and meta-*C*) or individual estimates obtained from a single-subject Bayesian model fit (M-ratio and log M-ratio). Larger non-transparent dots and corresponding leftmost values represent the mean values across participants (superiority rating, *d’*, *C*, and meta-*C*) or the group-level hierarchical Bayesian maximum a posteriori (MAP) probability estimates (M-ratio and log M-ratio). Error bars represent 95% confidence intervals of the mean (superiority rating, *d’*, *C*, and meta-*C*) or 95% highest-density intervals (HDI) of posterior distributions (M-ratio and log M-ratio). The rightmost values indicate statistical test values. Asterisks represent statistical significance (* *p* < 0.05, **** *p* < 0.0001). M-ratio = meta-*d’* / *d’*. log M-ratio = log(meta-*d’* / *d’*). VAS, visual analog scale. a.u., arbitrary unit

We asked participants to rate their superiority/inferiority compared to an average peer. The mean superiority rating score was 0.082, significantly greater than zero (*t*_*36*_ = 2.633, *p* = 0.012, Cohen’s *d* = 0.433 [95% CI: 0.099, 0.766]), confirming the superiority bias of the participants toward their own abilities or traits (Fig. 2A).

### Ponzo illusion

Figure 2B presents the SDT parameter estimates for the Ponzo illusion. One-sample t-tests indicated significantly positive *d’* values under control conditions (*t*_*36*_ = 18.707, *p* = 3.953 × 10^−20^, Cohen’s *d* = 3.075 [95% CI: 2.742, 3.409]), while *d’* was comparable to zero under depth cue conditions (*t*_*36*_ = 0.905, *p* = 0.371, Cohen’s *d* = 0.149 [95% CI: − 0.185, 0.482]). Criterion *C* was comparable to zero under both control (*t*_*36*_ = 0.360, *p* = 0.721, Cohen’s *d* = − 0.059 [95% CI: − 0.393, 0.274]) and depth cue (*t*_*36*_ = 1.560, *p* = 0.127, Cohen’s *d* = − 0.257 [95% CI: − 0.590, 0.077]) conditions. In addition, criterion meta-*C* was comparable to zero under both control (*t*_*36*_ = 0.689, *p* = 0.495, Cohen’s *d* = − 0.113 [95% CI: − 0.447, 0.220]) and depth cue (*t*_*36*_ = 0.548, *p* = 0.587, Cohen’s *d* = 0.090 [95% CI: − 0.243, 0.423]) conditions.

For type 2 M-ratio and log M-ratio estimates, we performed a hierarchical Bayesian estimation of metacognitive parameters from confidence ratings [41]. The group-level hierarchical Bayesian maximum a posteriori probability (MAP) M-ratio estimates were 0.744 and 0.628 (control and depth cue conditions, respectively). They were smaller than one under both control (95% HDI: 0.650, 0.842) and depth cue (95% HDI: 0.473, 0.772) conditions. Log M-ratio MAP estimates were − 0.292 and − 0.432 (control and depth cue conditions, respectively). They were smaller than zero under both control (95% HDI: − 0.425, − 0.167) and depth cue (95% HDI: − 0.736, − 0.249) conditions, indicating that metacognitive monitoring is not optimal for either task.

Figure 2C shows the between-condition differences (depth cue − control) in the SDT parameter estimates for the Ponzo illusion. Under depth cue conditions, smaller *d’* values could be obtained than under control conditions (*t*_*36*_ = 11.042, *p* = 4.126 × 10^−13^, Cohen’s *d* = − 1.815 [95% CI: − 2.149, − 1.482]), confirming that the depth cues induced a strong Ponzo illusion. Criteria *C* and meta-*C* were comparable between the depth cue and control conditions (*C*, *t*_*36*_ = 1.699, *p* = 0.098, Cohen’s *d* = − 0.279 [95% CI: − 0.613, 0.054]; meta-*C*, *t*_*36*_ = 1.255, *p* = 0.218, Cohen’s *d* = 0.206 [95% CI: − 0.127, 0.539]). We did not find meaningful between-condition differences for the M-ratio (MAP = − 0.105 [95% HDI: − 0.279, 0.052]) or log M-ratio (MAP = − 0.137 [95% HDI: − 0.437, 0.079]), indicating comparable metacognitive performance between the conditions. Note that mean confidence (metacognitive bias) was significantly smaller for the depth cue condition compared to the control condition (2.829 vs 3.070, *t*_*36*_ = 5.969, *p* = 7.638 × 10^−7^, Cohen’s *d* = − 0.981 [95% CI: − 1.315, − 0.648]).

### Correlations between superiority rating, perceptual sensitivity, and metacognitive performance

**Fig. 3:**
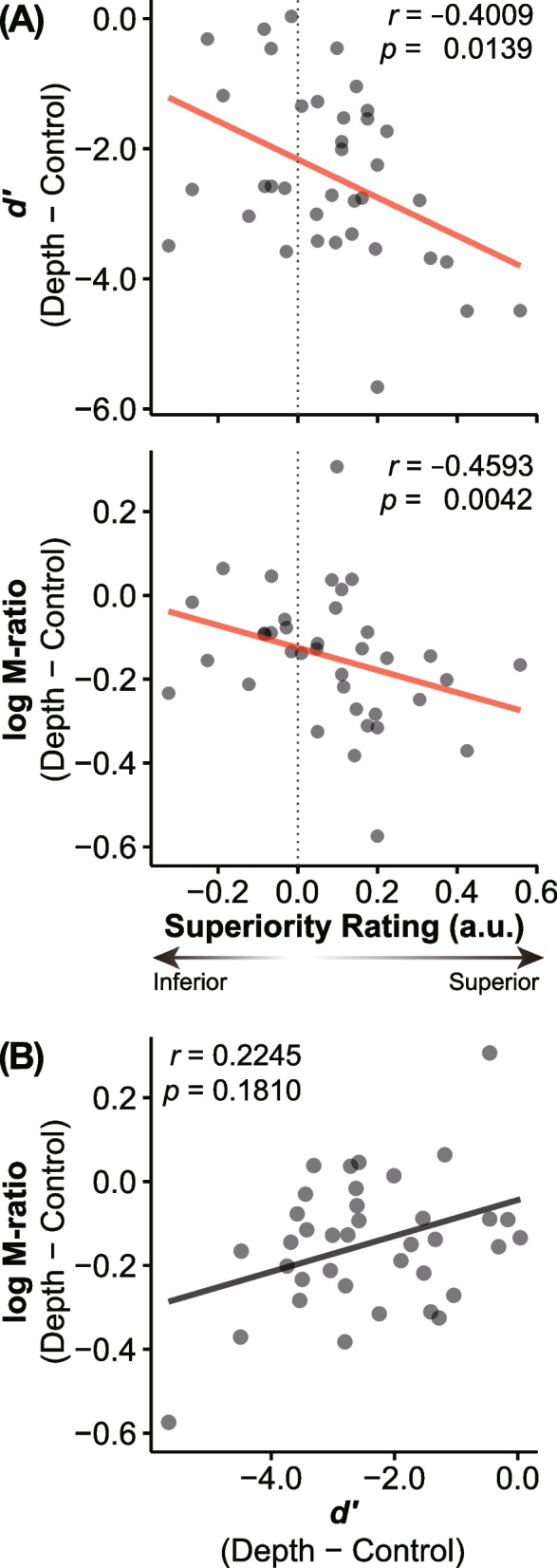
Correlations between superiority rating, perceptual sensitivity (*d’*), and metacognitive efficiency scores (log M-ratio). **A** Both the *d’* value and log M-ratio exhibited significant correlations with superiority rating scores. **B** No significant correlation between *d’* and log M-ratio. Transparent dots represent individual data points. Transparent lines represent linear regression fit using ordinary least squares. a.u., arbitrary unit

Figure 3 shows correlations between superiority ratings, perceptual sensitivity (*d’*), and metacognitive performance (log M-ratio) scores. Both *d’* (*rho* = − 0.401 [95% CI: − 0.642, − 0.088], *p* = 0.014) and log M-ratio (*rho* = − 0.459 [95% CI: − 0.682, − 0.159], *p* = 0.004) significantly correlated with superiority rating scores (Fig. 3A), while no significant correlation could be found between the *d’* value and log M-ratio (Fig. 3B*, rho* = 0.225 [95% CI: − 0.107, − 0.511], *p* = 0.181). These results remained constant even when controlling for each other and for age. We detected significant partial correlations between *d’* and superiority rating scores (*rho*_*p*_ = − 0.344 [95% CI: − 0.601, − 0.022], *p* = 0.039) and between log M-ratio and superiority rating scores (*rho*_*p*_ = − 0.414 [95% CI: − 0.650, − 0.104], *p* = 0.012) while controlling for the log M-ratio and *d’*, respectively. We found significant partial correlations between *d’* and superiority rating scores (*rho*_*p*_ = − 0.376 [95% CI: − 0.624, − 0.059], *p* = 0.024) and between log M-ratio and superiority rating scores (*rho*_*p*_ = − 0.500 [95% CI: − 0.709, − 0.211], *p* = 0.002) while controlling for age.

We found nonsignificant correlations between criteria measures and the superiority rating scores (*C*, *rho* = − 0.172 [95% CI: − 0.470, 0.161], *p* = 0.309; meta-*C*, *rho* = − 0.206 [95% CI: − 0.497, 0.127], *p* = 0.222), indicating that decision criteria were not associated with superiority rating. Furthermore, there was no significant correlation found between metacognitive bias and superiority rating scores (*rho* = 0.058 [95% CI: − 0.271, 0.375], *p* = 0.731). We observed no significant correlations between superiority rating and SDT parameters in the control condition (*d’*, *rho* = − 0.160 [95% CI: − 0.173, 0.460], *p* = 0.345; *C*, *rho* = − 0.125 [95% CI: − 0.431, 0.208], *p* = 0.462; meta-*C*, *rho* = 0.119 [95% CI: − 0.214, 0.426], *p* = 0.487; log M-ratio, *rho* = 0.014 [95% CI: − 0.311, 0.337], *p* = 0.931), suggesting that depth cue was a significant factor.

One might argue that our same/different task may bias participants toward one or the other alternative, affecting their metacognitive performance. However, we did not find a significant correlation between criterion C and log M-ratio (*rho* = − 0.300 [95% CI: − 0.026, − 0.569], *p* = 0.071). In addition, as hierarchical Bayesian procedures shrink inter-individual variability within a group, it is possible that parameter estimates from single-subject fits fail to capture accurate relationships. We thus performed hierarchical Bayesian estimation with simultaneous regression with SI as a covariate and confirmed a significant correlation with log M-ratio (*rho* = − 0.792 [95% CI: − 0.888, − 0.630], *p* = 5.218 × 10^−9^).

### Machine learning model

**Fig. 4:**
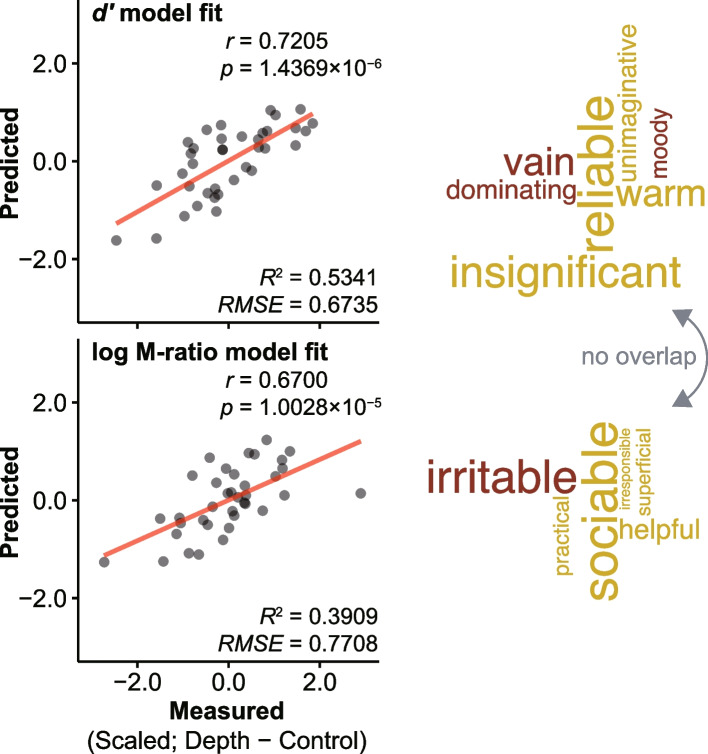
Machine learning prediction of perceptual sensitivity (*d’*) and metacognitive efficiency (log M-ratio) from superiority rating scores. Relaxed elastic net regression with leave-one-sample-out cross-validation created prediction models for *d’* (top row) and log M-ratio (bottom row) from superiority rating scores. Although the two models displayed similar prediction accuracy (left column), they consisted of different superiority rating items (right column). For more information, refer to Table 1. Transparent dots represent individual data points. Transparent lines represent linear regression fit using ordinary least squares. The word size was scaled relative to the (absolute value of) machine learning feature importance in the word cloud plot. Red and yellow words denote positive and negative feature importance, respectively (Table 1). *R*^*2*^, r-squared. *RMSE*, root-mean-square error

The relaxed elastic net regression with LOOCV revealed that different sets of superiority rating items predicted each SDT parameter estimate (Table 1). Both the *d’* and log M-ratio models achieved good accuracy (*d’*, *rho* = 0.721 [95% CI: 0.517, 0.847], *p* = 1.437 × 10^−6^, *R*^*2*^ = 0.534, *root-mean-square error* [*RMSE*] = 0.674; log M-ratio, *rho* = 0.670 [95% CI: 0.442, 0.817], *p* = 1.003 × 10^−5^, *R*^*2*^ = 0.391, *RMSE* = 0.771) and consisted of seven and six superiority rating items (Fig. 4 top and bottom row), respectively. No overlap could be observed between the two model items, indicating that the *d’* and log M-ratio parameter estimates were independently correlated (at least in part) with superiority ratings.

**Table 1:** Feature importance in machine learning models for predicting perceptual sensitivity (*d’*) and metacognitive efficiency (log M-ratio) based on superiority rating scores

Item		Feature importance
*d’*		
#29	vain	0.203
#21	dominating	0.128
#05	moody	0.107
#07	unimaginative	-0.119
#08	warm	-0.194
#13	insignificant	-0.237
#35	reliable	-0.266
*log M-ratio*		
#19	irritable	0.299
#48	irresponsible	-0.062
#33	superficial	-0.101
#32	practical	-0.116
#50	helpful	-0.159
#45	sociable	-0.323

Note that response and predictor variables included in the machine learning models were standardized for each variable, so the model feature importance should be interpreted accordingly. For prediction performance, see Fig. 4

### Latent architecture underlying machine learning model items

Given that the machine learning models selected different items for each model, it is possible that *d’* and log M-ratio were independently correlated with superiority ratings. However, an identical latent component might underlie the correlations even if the two models contained different items. To examine this possibility, we performed a PCA on 52-item superiority ratings and then assessed the relative contribution of each PC to each model.

The PCA with parallel analysis [51] revealed three significant PCs underlying the 52-item superiority ratings (Table 2). PC1 consisted of items such as “sociable” and “reliable,” so we labeled this PC as the “*extraversion*” component. PC2 consisted of items such as “persistent” and “honest”; this PC might thus reflect the “*honesty-humility*” component. PC3 consisted of items such as “sentimental” and “irritable”; thus, we regarded this PC as the “*neuroticism*” component.

**Table 2:** Principal component analysis (PCA) loadings for 52 superiority rating items

Item		PC1 (Extraversion)	PC2 (Honesty-humility)	PC3 (Neuroticism)
#45	sociable^c^	**0.406**	−0.117	**0.221**
#31^a^	unreliable	**0.398**	0.098	-0.042
#23^a^	unsociable	**0.389**	−0.178	0.142
#46^a^	unhappy	**0.375**	−0.084	0.135
#52^a^	unpopular	**0.346**	−0.039	−0.100
#11^a^	boring	**0.341**	−0.021	−0.021
#13^a^	insignificant^b^	**0.315**	−0.074	0.087
#35	reliable^b^	**0.307**	0.081	0.002
#14	good natured	**0.306**	**−0.240**	0.116
#44	determined	**0.305**	−0.047	0.069
#02^a^	frivolous	**0.299**	0.149	0.007
#15	humorous	**0.273**	−0.079	−0.072
#01	shrewd	**0.266**	−0.054	−0.121
#49	discriminating	**0.263**	0.041	−0.124
#03^a^	humorless	**0.256**	−0.107	−0.124
#20	happy	**0.259**	**−0.243**	0.132
#16	important	**0.257**	−0.003	0.019
#43	industrious	0.211	**0.346**	0.006
#10^a^	wasteful	0.035	**0.326**	0.103
#04	persistent	**0.289**	**0.312**	0.111
#48^a^	irresponsible^c^	**0.280**	**0.290**	0.102
#40^a^	impulsive	−0.145	**0.267**	−0.018
#42	honest	0.104	**0.258**	−0.025
#34^a^	critical	0.036	**0.244**	−0.052
#06	serious	0.076	**0.217**	**0.198**
#21^a^	dominating^b^	−0.115	**0.215**	−0.126
#24^a^	sentimental	0.138	0.137	**−0.259**
#17	calm	0.041	0.104	**−0.246**
#51	tolerant	−0.001	0.062	**−0.215**
#07^a^	unimaginative^b^	0.182	−0.139	**−0.211**
#12^a^	wavering	0.156	−0.155	**−0.202**
#27	imaginative	0.174	−0.154	**−0.201**
#38^a^	clumsy	0.203	0.066	**−0.200**
#22	skillful	0.156	−0.006	**−0.192**
#26	cautious	−0.139	0.187	**−0.176**
#19^a^	irritable^c^	0.017	0.081	**−0.176**
#25^a^	pessimistic	0.244	−0.116	**−0.162**
#47	intelligent	0.118	0.024	−0.114
#39^a^	unintelligent	0.173	0.081	−0.109
#28^a^	foolish	0.171	0.098	−0.082
#29^a^	vain^b^	−0.001	0.159	−0.077
#36	submissive	−0.125	−0.004	−0.069
#33^a^	superficial^c^	0.217	0.146	−0.031
#30	sincere	0.203	0.073	−0.026
#18^a^	squeamish	0.134	−0.039	−0.019
#41	popular	0.167	−0.069	−0.016
#32	practical^c^	0.215	0.129	−0.002
#09	modest	−0.084	0.188	0.066
#08	warm^b^	0.215	0.056	0.067
#05^a^	moody^b^	0.036	0.197	0.117
#50	helpful^c^	0.172	0.101	0.122
#37^a^	dishonest	0.143	0.179	0.148
Proportion of variance		0.276	0.138	0.090

Bold values represent absolute PC scores above 0.25 (PC1), above 0.2 (PC2), and above 0.15 (PC3)

^a^Reverse-coded items in superiority rating

^b^Perceptual sensitivity (*d’*) model items

^c^Metacognitive efficiency (log M-ratio) model items. For more information, see Table 1 and Fig. 4

**Fig. 5:**
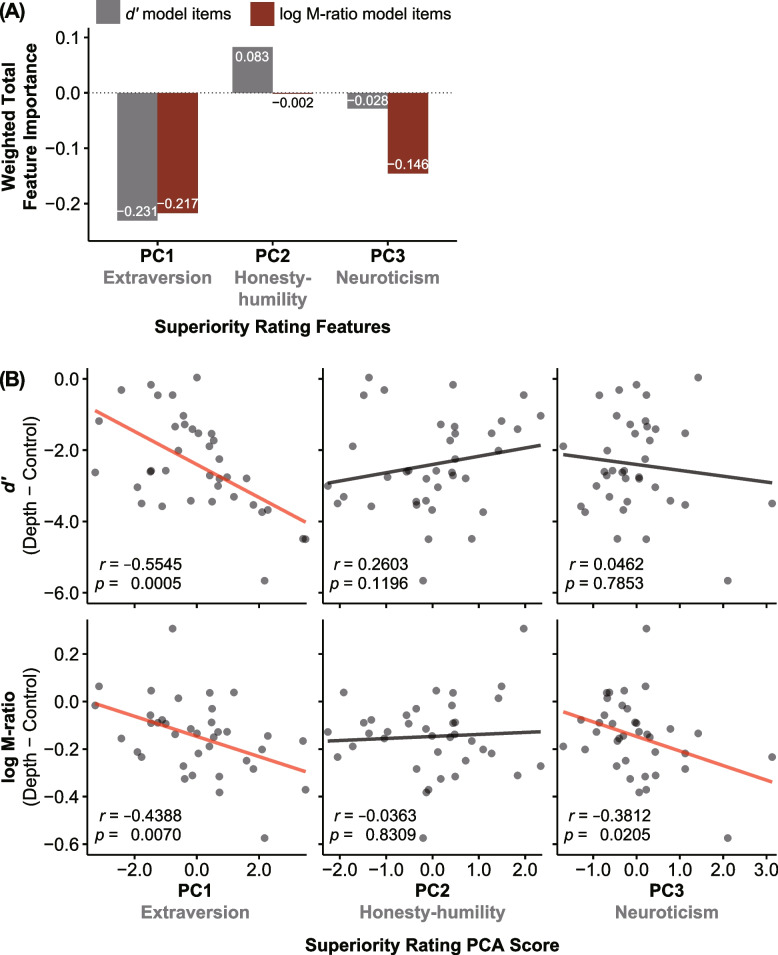
Latent relationship between the superiority illusion and the Ponzo illusion. **A** Weighted total feature importance values (the products of machine learning feature importance and PCA loadings) between the models were comparable in PC1 but dissociable in PC2 and PC3. The results indicate that codes related to SI were overlapping yet dissociable between the Ponzo illusion magnitude (*d’*) and metacognitive performance (log M-ratio). **B** Generic (machine learning irrelevant) relationships between the three PCA scores and *d’* (top row) and between the three PCA scores and log M-ratio (bottom row). Transparent dots represent individual data points. Transparent lines represent linear regression fit using ordinary least squares

Figure 5A presents the weighted total feature importance (machine learning feature importance (Table 1) × PCA loadings for the superiority rating items (Table 2)) for each PC and model. The PC1 importance was comparable between the *d’* and log M-ratio model items. However, the *d’* and log M-ratio model items weigh more on PC2 and PC3, respectively. Furthermore, we confirmed that interindividual correlations follow a similar “common yet dissociable” pattern between PCs and SDT parameter estimates (Fig. 5B). We further confirmed the generic (machine learning irrelevant) relationships between the PCs and SDT measures. The correlation between *d’* and superiority rating PCA score was significant in PC1 (*rho* = − 0.555 [95% CI: − 0.745, − 0.281], *p* = 0.0005), while no significant correlations were found in PC2 (*rho* = 0.260 [95% CI: − 0.070, 0.539], *p* = 0.119) and PC3 (*rho* = 0.046 [95% CI: − 0.282, 0.365], *p* = 0.785) (Fig. 5B top row). The correlations between the log M-ratio and superiority rating PCA score were significant in PC1 (*rho* = − 0.439 [95% CI: − 0.668, − 0.134], *p* = 0.007) and PC3 (*rho* = − 0.381 [95% CI: − 0.628, − 0.065], *p* = 0.021). In contrast, no significant correlation was found in PC2 (*rho* = − 0.036 [95% CI: − 0.356, 0.291], *p* = 0.831) (Fig. 5B bottom row).

## Discussion

Using hierarchical Bayesian estimation and machine learning of SDT measures, we aimed to determine how SI influences Ponzo illusion magnitude and metacognitive performance. SI of oneself over an average peer is suggestively crucial for a healthy mental state and behavior [4, 52]. However, whether such SI involves low-level perceptual tasks has remained elusive. Our behavioral results revealed that SI correlated with Ponzo illusion magnitude and metacognitive ability. Next, cross-validated regularized regression (relaxed elastic net) further uncovered the latent architecture behind them. Ponzo illusion magnitude and metacognitive performance were influenced by the same superiority feature (extraversion), while they were affected by the other distinct superiority features (honesty-humility and neuroticism, respectively). Perception and metacognition are thus liable to influences from overlapping and separable superiority features. SI might have various psychological benefits [3, 4, 6–10] and exert concurrent biasing effects on Ponzo illusion perception and metacognition, perhaps due to its illusory and self-affirmative belief.

Our findings are in good agreement with recent studies suggesting that global (i.e., general self-belief) and local (i.e., trial-wise decision evaluation) metacognition closely interact, forming a hierarchical structure that impacts mental health [53–55]. They suggested that global self-beliefs bias local confidence, while local confidence helps form global self-beliefs. SI and trial-wise metacognition were closely related, perhaps because the hierarchical structure embeds them as reciprocally connected layers. SI might accordingly exert a top–down influence on within-hierarchy local metacognition while simultaneously biasing Ponzo illusion strength via a different route, proven by the dissociable contributions of SI features to Ponzo illusion magnitude and local metacognitive performance.

The self-affirmative SI features contributed to perceptual and metacognitive performance. Human variation in subjective superiority in each feature might reflect one’s belief (or priority) of being superior in a given domain [19], eventually forging individual differences in behavioral heuristics that regulate diverse information processing layers. Humans striving to maintain positive self-regard might be a significant source of top-down bias for perceptual capacity to handle contextual information (i.e., the degree to incorporate contexts into visual percepts) and metacognitive ability to monitor self-performance (i.e., the degree of illusory confidence in one’s perceptual ability). It is important to note that there are certain constructs that resemble SI, namely self-esteem, positive illusions, and optimism bias. Although their interrelationships are not yet fully understood and are beyond the scope of our study, some of their sub-dimensions might have a similar effect on the strength of the Ponzo illusion and/or metacognitive performance, as seen in SI.

We identified the *three* features of SI using trait words derived from Rosenberg et al. [42]. The authors suggested that there were *two* primary components underlying personality impression (competence and warmth) [56]; our results thus appeared to be inconsistent with theirs regarding the number of dimensions. However, *the impression of others* and *the assessment of one’s traits* might be different things. When people judge social groups, warmth and competence evaluations negatively correlate [57], implying a simplified judgment. Furthermore, Beer and Watson [58] described the convergence tendency of trait dimensions in peer ratings compared to self-ratings. These findings suggest that people use heuristics and judge others based on simplified trait structures. In other words, people might make scrupulous, albeit self-serving, appraisals of their characteristics, resulting in judgments based on elaborated trait structures [59].

Our findings demonstrated shared, yet dissociable, influences of SI on perceptual and metacognitive performance. Extraversion (PC1) is a core feature affecting *both* visual perception and metacognition, while others do not. Subjective superiority in extraversion was predictive of Ponzo illusion magnitude and metacognitive ability, possibly via lower sensitivity [60–62] and overconfidence [63, 64], respectively. However, lower sensitivity and overconfidence might not be as disparate as it first seems. They could reflect the two sides of the same coin as in the case of the Dunning–Kruger effect [52, 65], indicating poor performers’ overestimation of their ability [66–68].

Furthermore, honesty-humility (PC2) and neuroticism (PC3) impacted *either* Ponzo illusion strength or metacognitive performance, but not both. However, the difference between their contribution to the predictive models was striking. While honesty-humility was predictive of Ponzo illusion magnitude, consistent with the findings showing the correlation between honesty-humility and less dependence on contextual information [69, 70], it contributed to the prediction model relatively weakly (Fig. 5A). Instead, neuroticism contributed to the prediction model more substantially, approximately twice as much as honesty-humility. Therefore, neuroticism might be more operative than honesty-humility in dissociating superiority features and behavioral performance. It is well known that neuroticism exhibits fundamental roles in a wide array of health and life outcomes [71]. Our findings are in line with recent studies suggesting that anxiety and depression, which are highly linked to neuroticism [72, 73], are closely associated with metacognition but not first-order task performance [74, 75].

In conclusion, SI correlated with Ponzo illusion strength and metacognitive performance. Moreover, using cross-validated regularized regression, we unveiled their latent architecture predictive of Ponzo illusion perception and metacognition. A significant limitation of our study is that we did not incorporate other classes of visual illusion. How SI influences behavior might hinge on the illusion type [32]. In addition, we did not perform a priori sample size determination, and the present findings potentially do not generalize to females as we included only male participants. However, a recent meta-analysis showed that SI per se is constant across gender groups [9]. Another limitation may be that our experiment employed the same/different task instead of a 2IFC task (becoming common in the field) because these two task variants might involve different cognitive processes [76]. Although further research is warranted to resolve these issues, we suggest that SI is a cardinal cognitive bias that involves a vast assortment of behaviors as an illusion is imperative for humans to somehow thrive in a world of ambiguity.

## Data Availability

Data and code required to reproduce the results in this paper are found at https://github.com/dicemt/matsuyoshi_ponzo_metacognition.
